# Comparative Evaluation of Uncorrected Visual Acuity in Refractive Error Patients Aged Between 18-25 Years and 38-45 Years: A Cross-Sectional Study

**DOI:** 10.7759/cureus.112461

**Published:** 2026-07-11

**Authors:** Debapriyo Chatterjee, Shoubhik Chakraborty, Anushka Sain, Sahanaz Hossain, Biswajit Mondal, Riddhi S Dwibedi, Bidisha Bhattacharya, Ananya Choudhurya

**Affiliations:** 1 Optometry, New School Human Management (NSHM) College of Management and Technology, Durgapur, IND; 2 Public Health, New School Human Management (NSHM) College of Management and Technology, Durgapur, IND; 3 Ophthalmology, Kalinga Institute of Medical Sciences, Bhubaneswar, IND

**Keywords:** compound myopic astigmatism, logmar, myopia, refractive error, uncorrected visual acuity

## Abstract

Background

Refractive error is a widespread cause of decreased visual acuity, which can impact academic success, work productivity, and quality of life. Uncorrected visual acuity (UCVA) provides baseline information regarding refractive status before optical correction. The purpose of this study was to compare UCVA between adults aged 18-25 years and 38-45 years.

Methodology

This cross-sectional study was conducted in the Department of Optometry at the NSHM College of Management and Technology, Durgapur, West Bengal, India, between November 2025 and April 2026. A total of 300 participants undergoing refractive assessment were enrolled, comprising two equal age groups. Group A consisted of 150 individuals aged 18-25 years, and Group B consisted of 150 individuals aged 38-45 years. UCVA was measured using a Snellen chart and subsequently converted to logMAR values for statistical analysis. Each participant underwent a comprehensive refraction assessment and was included in the study only if their best-corrected visual acuity was 6/9 or better. Welch’s independent-samples t-test was applied to compare UCVA between the groups, while categorical variables were analyzed using the chi-square test.

Results

The mean UCVA for the right eye was significantly poorer in the 18-25-year group than in the 38-45-year group (0.249 ± 0.199 vs. 0.142 ± 0.117 logMAR; mean difference: 0.107; 95% confidence interval (CI): 0.070-0.144; p < 0.001). Left eye UCVA was poorer in the younger group (0.187 ± 0.199 vs. 0.136 ± 0.120 logMAR; mean difference: 0.051; 95% CI: 0.014-0.088; p = 0.008). There was no significant difference in gender distribution between the groups (p = 0.160). The distribution of refractive errors varied significantly between the groups (p < 0.001), and compound myopic astigmatism was more prevalent in the 18-25-year group (149 eyes, 49.7%) compared with the 38-45-year group (1 eye, 0.3%).

Conclusions

The UCVA scores of participants aged 18-25 years were significantly lower than those of the 38-45-year-old participants. The poorer UCVA was mainly associated with higher compound myopic astigmatism, highlighting the need for regular refractive screening and timely optical correction among young adults.

## Introduction

Vision is one of the most important sensory modalities and plays a crucial role in daily functioning and quality of life. Refractive error is one of the most prevalent and treatable causes of visual impairment worldwide and remains a major cause of avoidable vision loss. Suboptimal vision correction is available and widely used, but many people still experience visual impairment due to factors such as delayed diagnosis, lack of awareness, and infrequent eye exams [1-3].

Uncorrected visual acuity (UCVA) is a person’s visual acuity without refractive correction. It is one of the most important indicators of the visual system’s working capacity during clinical diagnosis without glasses or contact lenses. UCVA is a useful baseline assessment of the visual impact of refractive errors in optometric practice and an aid to comprehensive refractive assessment and the prescription of an appropriate optical correction [4,5].

Although age-related physiological changes occur in the crystalline lens and accommodative system, these changes primarily affect near vision and are characteristic of presbyopia rather than refractive error. In contrast, distance UCVA is primarily influenced by the type and magnitude of refractive error. While numerous studies have investigated age-related changes in accommodation and the epidemiology of refractive errors, few studies have directly compared distance UCVA between younger adults (18-25 years) and middle-aged adults (38-45 years) with refractive errors. This gap in the literature motivated the present investigation [6-8].

Understanding age-related differences in distance UCVA may provide valuable information for refractive assessment, timely optical correction, and vision screening. However, evidence directly comparing distance UCVA between young adults (18-25 years) and middle-aged adults (38-45 years) remains limited. Therefore, the present study was designed to determine whether clinically relevant differences in distance UCVA exist between these two age groups undergoing refractive assessment, without presuming the direction of the association. The findings may help guide age-specific refractive assessment, timely optical correction, and preventive eye care strategies.

## Materials and methods

Study design, setting, and period

A cross-sectional comparative study was conducted to evaluate and compare distance UCVA among individuals undergoing refractive assessment. Participants were categorized into the following two predefined age groups: 18-25 years (Group A) and 38-45 years (Group B). These age groups were selected based on clinically relevant stages of adult visual physiology and previous evidence describing age-related changes in accommodation [6-8]. Group A represented young adults with stable accommodative function, whereas Group B represented adults approaching the early presbyopic period, during which physiological changes in accommodation begin. This grouping enabled comparison between two clinically distinct stages of adult visual function. The study was conducted in the Department of Optometry at the NSHM College of Management and Technology, Durgapur. The study was conducted from November 2025 to April 2026. Individuals undergoing refractive assessment in the Optometry Outpatient Department during the study period constituted the study population.

Sample size

The sample size was determined to compare the mean UCVA between two independent age groups using the formula n = 2(Zα/2 + Zβ)²σ²/d². Based on an expected difference of 0.08 logMAR units between groups, a 95% confidence level, and 80% statistical power, the minimum required sample size was approximately 150 participants per group. Therefore, a total of 300 participants were recruited and equally allocated into Group A (18-25 years) and Group B (38-45 years).

Inclusion and exclusion criteria

Individuals undergoing refractive assessment with a best-corrected visual acuity (BCVA) of 6/9 or better and belonging to either the 18-25-year or the 38-45-year age group were eligible for inclusion, irrespective of refractive status. Participants who provided written informed consent were included in the study.

Patients were excluded if they had any ocular disease, including cataract, glaucoma, retinal disease, media opacity, active ocular infection, or ocular inflammation, that could affect visual acuity assessment. Patients who had undergone ocular surgery or had systemic diseases affecting vision, such as diabetes mellitus, were excluded from the study.

Study procedure

Participants were asked to read a standard Snellen visual acuity chart (Appasamy Associates, Chennai, India) at a distance of 6 m under proper illumination. Visual acuity was recorded using standard clinical procedures and subsequently converted to logMAR notation for statistical analysis [4]. Care was taken in measuring visual acuity, which was performed without spectacles or contact lenses. Participants were categorized into two predefined age groups based on clinically relevant stages of adult visual physiology and previous evidence describing age-related changes in accommodation [6-8]. Individuals aged 18-25 years represented young adults with relatively stable accommodative function, whereas those aged 38-45 years represented adults approaching the early presbyopic period, during which physiological changes in accommodation begin. This grouping was selected to facilitate comparison between two clinically distinct stages of adult visual function rather than to evaluate a continuous age-related association.

Refraction was used to verify the existence of a refractive error after UCVA measurement. A streak retinoscope (Heine Optotechnik, Herrsching, Germany) was used for objective refraction, followed by subjective refinement to ascertain the participant’s refractive status and the Duo-chrome test’s refraction endpoint [5]. Following refractive correction, the BCVA was measured. Only individuals with a BCVA of 6/9 or better were included to ensure that reduced unassisted vision was mostly due to refractive error rather than ocular illness.

Based on age, the participants were split into two groups. Individuals in Group B were between the ages of 38 and 45, whereas those in Group A were between the ages of 18 and 25. To determine whether the UCVA pattern differed between younger and older persons, the UCVA results of both groups were compared.

Outcome measure

The primary outcome measure was UCVA. UCVA findings were compared between the 18-25-year and 38-45-year age groups.

Statistical analysis

Data were entered into Microsoft Excel (Microsoft Corp., Redmond, WA, USA), and data completeness and accuracy were assessed before analysis. Demographic data and visual acuity were summarized using descriptive statistics. Continuous variables are presented as mean ± standard deviation, whereas categorical variables are presented as frequencies and percentages. The Shapiro-Wilk test was performed to assess the normality of UCVA logMAR values. Although the normality test indicated a non-normal distribution (p < 0.001), Welch’s independent-samples t-test was applied. Welch’s independent-samples t-test was selected because it does not assume equal variances between groups and provides more reliable estimates when variances are heterogeneous. In addition, given the large sample size (n = 150 per group), Welch’s t-test is robust to moderate departures from normality under the Central Limit Theorem. Effect sizes were calculated using Cohen’s d. The chi-square test was used to compare categorical variables such as gender distribution and refractive error patterns between age groups. Statistical significance was set at p-values <0.05. Refractive error distribution was analyzed on an eye-wise basis, whereas demographic and UCVA comparisons were performed at the participant level.

Ethical considerations

The investigation was conducted in accordance with ethical principles for clinical research. Ethical approval was obtained from the Institutional Ethics Committee of NSHM College of Management and Technology (approval number: NCMT/RC/2025/OPT/006). All subjects gave their informed consent before data collection. Participants were free to leave the study at any time, and no one was coerced into participating. The participants’ personal data were kept confidential and used only for the study.

## Results

In the present study, 300 participants were included, equally distributed into two age groups: 18-25 years (150 participants) and 38-45 years (150 participants). The distance UCVA values were converted to logMAR units for statistical analysis. Table 1 shows the normality test results of the UCVA logMAR values for both eyes in the two age groups. The Shapiro-Wilk results were statistically significant (p < 0.001) in both groups for the right and left eye UCVA values, suggesting that the right and left eye UCVA values were not normally distributed.

**Table 1:** Shapiro–Wilk normality test for UCVA logMAR. *: Statistically significant at p-values <0.05. UCVA = uncorrected visual acuity

Variable	Age group	n	Shapiro–Wilk W	P-value
UCVA right eye logMAR	18–25 years	150	0.790	<0.001*
UCVA right eye logMAR	38–45 years	150	0.795	<0.001*
UCVA left eye logMAR	18–25 years	150	0.785	<0.001*
UCVA left eye logMAR	38–45 years	150	0.789	<0.001*

Table 2 presents a comparison of the UCVA logMAR scores of the 18-25-year age group with the scores of the 38-45-year age group. There were significant differences between the 18-25- and 38-45-year groups in the mean UCVA of the right eye (0.249 ± 0.199 and 0.142 ± 0.117, respectively). This difference was statistically significant (p < 0.001). Likewise, in the 18-25-year group, the mean UCVA of the left eye was poorer than that of the 38-45-year group, with mean logMAR values of 0.187 ± 0.199 and 0.136 ± 0.120, respectively. This difference was also statistically significant (p = 0.008).

**Table 2:** Comparison of UCVA between age groups. *: Statistically significant at p-values <0.05. UCVA = uncorrected visual acuity; SD = standard deviation; CI = confidence interval

Variable	18–25 years, mean ± SD	38–45 years, mean ± SD	Mean difference	95% CI	t	df	P-value	Cohen’s d
UCVA right eye logMAR	0.249 ± 0.199	0.142 ± 0.117	0.107	0.070 to 0.144	5.688	241.5	<0.001*	0.657
UCVA left eye logMAR	0.187 ± 0.199	0.136 ± 0.120	0.051	0.014 to 0.088	2.692	244.4	0.008*	0.311

**Figure 1:**
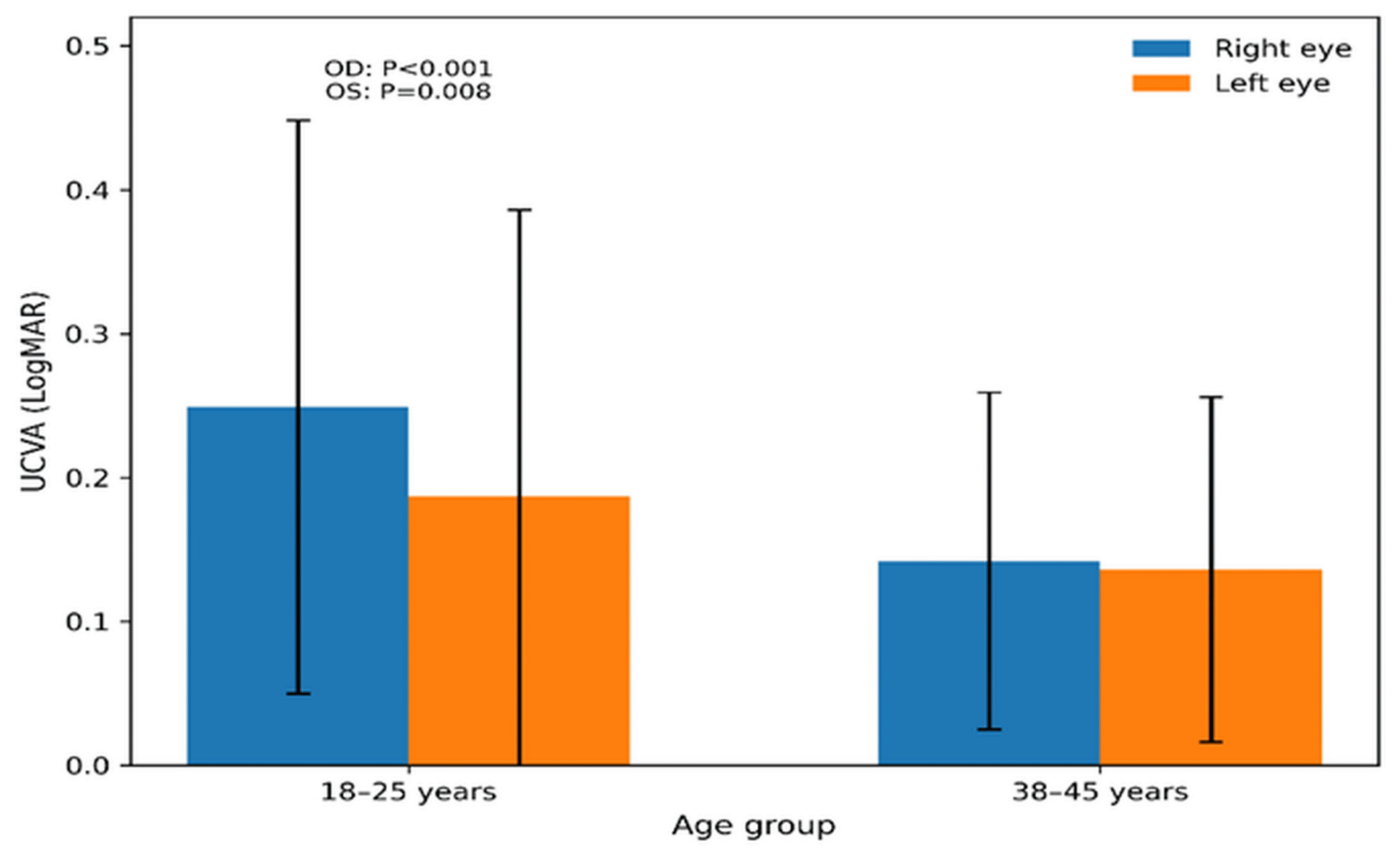
Comparison of uncorrected visual acuity logMAR values between the 18-25 and 38-45 age groups. The 18-25-year group showed significantly poorer UCVA in both the right and left eyes compared with the 38-45-year group. Error bars represent standard deviation. UCVA = uncorrected visual acuity

Figure 1 demonstrates that the 18-25-year age group had significantly higher logMAR values than the 38-45-year age group in both the right and left eyes. The differences were statistically significant for both eyes (OD: p < 0.001; OS: p = 0.008).

Table 3 shows the gender distribution between the two study groups. There were 81 males and 69 females in the 18-25-year age group. The 38-45-year age group had 93 males and 57 females. There was no statistically significant difference in gender distribution between the two groups, indicating they were comparable.

**Table 3:** Gender distribution between age groups. χ² = 1.970, df = 1, p = 0.160, Cramer’s V = 0.081.

Gender	18–25 years, n (%)	38–45 years, n (%)
Male	81 (54.0)	93 (62.0)
Female	69 (46.0)	57 (38.0)

Table 4 shows the eye-wise distribution of different types of refractive error in the two age groups. In the 18-25-year age group, compound myopic astigmatism (149 eyes, 49.7%) was the most common refractive error, followed by myopia (45 eyes, 15.0%) and simple myopic astigmatism (41 eyes, 13.7%). Simple myopic astigmatism was the most frequent form in the 38-45-year age group, with 123 (41.0%) cases, followed by plano/emmetropia (55 eyes, 18.3%), hyperopia (42 eyes, 14.0%), and myopia (39 eyes, 13.0%). There was a significant difference in the distribution of refractive error types between the two age groups (p < 0.001).

**Table 4:** Eye-wise refractive error distribution between age groups. χ² = 242.775, df = 7, p < 0.001*, Cramer’s V = 0.636. *: Statistically significant at a p-value <0.05.

Type of refractive error	18–25 years, n (%)	38–45 years, n (%)
Compound hyperopic astigmatism	8 (2.7)	9 (3.0)
Compound myopic astigmatism	149 (49.7)	1 (0.3)
Hyperopia	12 (4.0)	42 (14.0)
Mixed astigmatism	21 (7.0)	26 (8.7)
Myopia	45 (15.0)	39 (13.0)
Plano/Emmetropia	9 (3.0)	55 (18.3)
Simple hyperopic astigmatism	15 (5.0)	5 (1.7)
Simple myopic astigmatism	41 (13.7)	123 (41.0)

**Figure 2:**
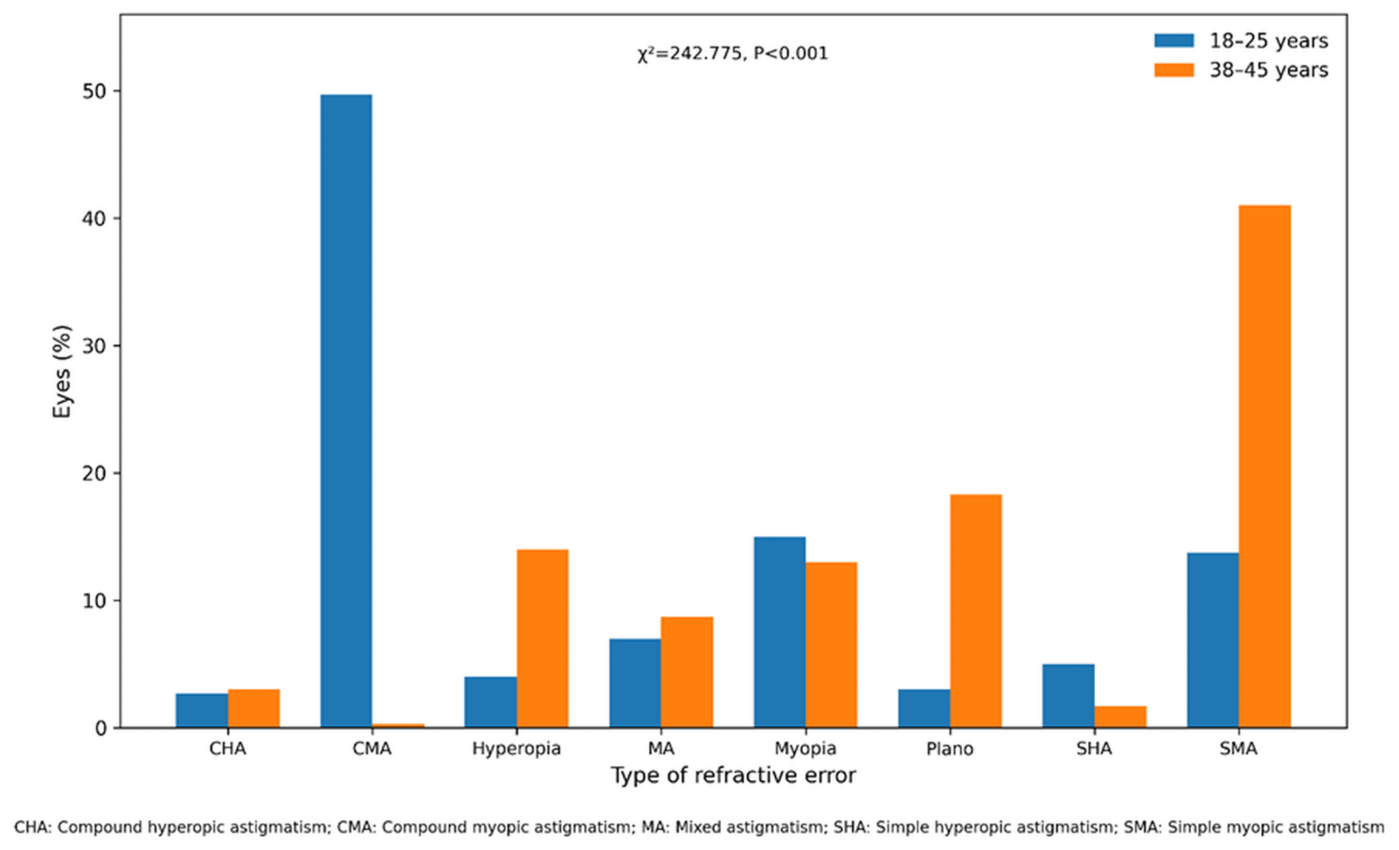
Eye-wise distribution of refractive error types between the 18-25 and 38-45 age groups. Compound myopic astigmatism was the predominant refractive error in the younger age group, whereas simple myopic astigmatism was more frequent in the 38-45-year group (123 eyes, 41.0%). CHA = compound hyperopic astigmatism; CMA = compound myopic astigmatism; MA = mixed astigmatism; SHA = simple hyperopic astigmatism; SMA = simple myopic astigmatism

Figure 2 illustrates a significant difference in the distribution of refractive error types between the two age groups (χ² = 242.775, p < 0.001). Compound myopic astigmatism was the predominant refractive error among participants aged 18-25 years (149 eyes, 49.7%).

The mean UCVA of the right eye was significantly poorer in the 18-25-year group than in the 38-45-year group (mean logMAR values: 0.249 ± 0.199 and 0.142 ± 0.117, respectively). The mean difference was 0.107 logMAR, which was statistically significant (95% confidence interval (CI): 0.070 to 0.144; t = 5.688, df = 241.5, p < 0.001), with a moderate effect size (Cohen’s d = 0.657). Similarly, logMAR values for the left eye were significantly worse in the 18-25-year group (0.187 ± 0.199) than in the 38-45year group (0.136 ± 0.120). The mean difference was 0.051 logMAR and was statistically significant (95% CI: 0.014-0.088; t = 2.692, df = 244.4, p = 0.008), with a small effect size (Cohen’s d = 0.311). There was no significant difference in the gender distribution among the groups (χ² = 1.970, p = 0.160). The distribution of refractive errors across the two age groups differed significantly (χ² = 242.775, p < 0.001).

The gender distribution was comparable between the two groups. There was no significant difference in gender distribution between the two age groups (χ² = 1.970, p = 0.160). Participants aged 18-25 years had significantly higher logMAR values than those aged 38-45 years in both the right and left eyes (OD: p < 0.001; OS: p = 0.008). The effect size was moderate for the right eye (Cohen’s d = 0.657) and small for the left eye (Cohen’s d = 0.311). The distribution of refractive error types differed significantly between the two age groups (χ² = 242.775, p < 0.001).

## Discussion

The present study compared distance UCVA between young adults (18-25 years) and middle-aged adults (38-45 years) undergoing refractive assessment. The findings demonstrated significantly higher logMAR values (poorer UCVA) in the younger age group than in the middle-aged group. The mean UCVA in the right eye was 0.249 ± 0.199 logMAR among participants aged 18-25 years compared with 0.142 ± 0.117 logMAR among those aged 38-45 years. Similarly, the mean UCVA in the left eye was 0.187 ± 0.199 logMAR in the younger group and 0.136 ± 0.120 logMAR in the older group. These findings indicate that younger adults experienced greater impairment in uncorrected distance vision than their older counterparts.

The poorer UCVA observed among younger adults may reflect the higher prevalence of myopic refractive errors, particularly compound myopic astigmatism, as reported in previous studies among medical students and young adults [9,10]. In addition, cohort-specific factors such as greater educational demands, prolonged near-work activities, and increased digital device use may have contributed to the observed differences rather than physiological aging alone [11-13]. Because the present study employed a cross-sectional design, these findings should not be interpreted as evidence of age-related changes in distance UCVA but rather as differences between two adult cohorts.

The higher prevalence of myopia among younger adults may be associated with increased academic demands, prolonged near work, and extensive digital device use. The International Myopia Institute has emphasized the role of environmental and behavioral factors in myopia development and progression [11]. Dutheil et al. reported a significant association between near-work activities and myopia [12]. Recent International Myopia Institute reports further highlighted the growing influence of modern visual habits on refractive development and the importance of myopia prevention strategies [13].

The distribution of refractive errors differed significantly between the two age groups. Participants aged 18-25 years demonstrated a predominance of compound myopic astigmatism, whereas those aged 38-45 years showed a greater proportion of simple myopic astigmatism, hyperopia, and plano/emmetropia. The relatively better UCVA observed among older participants may therefore be attributed to the lower prevalence of compound myopic astigmatism and the greater proportion of plano/emmetropic eyes. These findings should also be interpreted in the context of the increasing prevalence of myopia worldwide. Morgan et al. described myopia as a major public health concern with significant social and economic implications [14]. Pan et al. reported that myopia prevalence has increased substantially across different populations worldwide [15]. Similar concerns regarding the growing burden of myopia have been highlighted in subsequent publications [16].

Population-based studies from India have also demonstrated significant age-related variations in refractive error patterns [17]. Morgan et al. further emphasized the increasing prevalence of myopia and the need for effective preventive strategies [18]. Indian epidemiological studies have reported considerable variation in refractive error patterns across different age groups and geographical regions [19]. Holden et al. projected that nearly half of the world’s population may be myopic by 2050, underscoring the need for early identification and management of refractive errors [20].

The findings of the present study are also supported by evidence from student populations. Studies conducted among university and medical students have consistently reported a high prevalence of refractive errors, particularly myopia [21-22]. Furthermore, recent evidence suggests that increased digital screen exposure may contribute to myopia progression and visual stress among young individuals [23,24]. These observations are consistent with the present findings, in which younger adults demonstrated poorer UCVA and a greater burden of myopic refractive errors.

The findings of the present study have important implications for clinical optometry and community eye health. The poorer UCVA observed among young adults highlights the importance of routine refractive screening in this population. Early detection and timely correction of refractive errors may improve visual performance, academic productivity, and quality of life while reducing the burden of avoidable visual impairment. In addition, counseling regarding visual hygiene, including appropriate working distance, regular visual breaks, adequate illumination, and increased outdoor activities, may help reduce visual stress and support better visual outcomes.

A notable finding of the present study was the substantially higher proportion of compound myopic astigmatism in the younger age group compared with the middle-aged group. The refractive classifications and data entries were rechecked and verified against the original clinical records. Because the present study was cross-sectional and clinic-based, this difference should not be interpreted as evidence of an age-related change in astigmatism subtype. Rather, it may reflect differences in the refractive characteristics of the study cohorts or referral patterns. Further population-based longitudinal studies are required to determine whether this observation is reproducible.

The present study has several limitations. First, its cross-sectional design precludes causal inference and does not permit differentiation between true age-related changes and cohort effects. Second, participants were recruited from a single optometry outpatient department, which may limit the generalizability of the findings to the broader population. Third, although the marked difference in the prevalence of compound myopic astigmatism between the two age groups was verified against the original clinical records, this finding should be interpreted cautiously and confirmed in larger population-based studies. Finally, refractive error distribution was analyzed on an eye-wise basis; therefore, the potential non-independence of fellow eyes may have influenced the statistical estimates. Future multicenter longitudinal studies with population-based sampling and participant-level analyses are warranted to validate these findings.

Furthermore, because participants were recruited from a single optometry outpatient department rather than the general population, the findings primarily reflect a clinic-based population and should not be generalized to the wider community without caution.

## Conclusions

The present study revealed that the distance UCVA was significantly lower for the 18-25-year-olds compared to 38-45-year-olds for both right and left eyes. The difference was larger in the right eye with a medium-sized effect, but smaller in the left eye with a significant, but relatively small-sized effect. The poorer UCVA in the younger age group was mainly associated with the higher frequency of compound myopic astigmatism, which was the predominant refractive error in this group. Conversely, there was a higher number of simple myopic astigmatism, hyperopic, and plano/emmetropic eyes in the group aged between 38 and 45 years. The difference in UCVA was unlikely to be attributable to differences in gender distribution between the groups. The overall results suggest that young adults may be at a higher risk of poorer UCVA due to a greater burden of compound myopic astigmatism. Refractive screening, prompt optical correction, and counselling on visual hygiene to youth/adults with special emphasis on students and individuals engaged in prolonged near work and using digital devices is important. Further larger community-based studies and studies of other (longer-term) child and young person groups (such as those with measures of axial length, keratometry, near work hours, screen time, and outdoor activity) would help to gain a clearer understanding of refractive error patterns across different age groups.
